# Statins, commonly coprescribed drugs, and concomitant risk factors: A protective, neutral, or harmful association with common cancer types development: A 10-year multicentric retrospective lebanese study

**DOI:** 10.1097/MD.0000000000034562

**Published:** 2023-09-29

**Authors:** Issam G. Chalhoub, Rita T. Boulos, Yara G. Dagher, Sandra El Helou, Karen G. Haifa, Bachir Atallah, Fadi Nasr, Issam Kassab, Mirna N. Chahine

**Affiliations:** a Faculty of Medical Sciences, Lebanese University, Hadath, Lebanon; b Statistics, Lebanese University, Hadath, Lebanon; c Hematology-Oncology Department, Hotel Dieu de France, Achrafieh, Beirut, Lebanon; d National Center of Pharmacovigilance, Faculty of Pharmacy, Lebanese University, Hadath, Lebanon; e Basic Sciences Department, Faculty of Medical Sciences, Lebanese University, Hadath, Lebanon; f Foundation-Medical Research Institutes (F-MRI), Beirut, Lebanon/Geneva, Switzerland.

**Keywords:** bladder, cancer, cardiovascular diseases, colorectal, Lebanon, lung, statins

## Abstract

Elevated blood levels of low-density lipoprotein cholesterol are a major cardiovascular risk factor, and cholesterol-lowering drugs are among the most prescribed drugs worldwide. Cancer is the second leading cause of death after cardiovascular diseases. The relationship between cancer development and statins intake is controversial, and there are no clear studies in Lebanon and the Middle East concerning this topic. Hence, our study aimed to search for any possible association of statin intake as well as other medications (proton pump inhibitors [PPI], metformin, Aspirin, Angiotensin-Converting Enzyme inhibitors, and fenofibrate) with lung, colorectal cancer (CRC), and bladder cancer development in the Lebanese population. A retrospective study was performed on 709 subjects divided into 2 main groups: control (no cancer ± statin intake), and cases (either lung, or colorectal, or bladder cancer ± statin intake). Collected data included the age and gender of the patient, socioeconomic status, presence of cardiovascular disease and comorbidities, cancer risk factors, and the intake type, dose, and duration of statins. Bivariate, multivariate, and binary logistic analyses were enrolled. Out of 709 participants, 63.2% were males and 75% were cancer-positive (24.1%: lung cancer, 26.7%: CRC, 24.1%: bladder cancer). The overall intake of statins was not shown to significantly affect cancer development. However, a duration-response relationship was established between Simvastatin and lung cancer (odds ratio [OR]=1.208) as well as bladder cancer (OR=1.189). No significant association was found between each statin and CRC. Although PPIs intake was associated with a possibly harmful effect on lung cancer development (OR=3.42), it revealed a protective association with CRC development (OR=0.38). Other risk factors such as smoking and age were strongly associated (harmful) with lung and bladder cancer development. Physical inactivity and a family history of CRC were each associated with a harmful effect on CRC development. A harmful association with the development of lung and bladder cancer was found with the increasing duration of intake of Simvastatin. Other drugs such as PPIs and specific risk factors were also associated negatively or positively with the development of these 3 cancers. These findings should be validated by further investigations to guide clinicians on optimal treatment options for their patients.

## 1. Introduction

Cardiovascular diseases (CVDs) are the primary cause of death worldwide. According to the World Health Organization, around 17.9 million people died from CVDs in 2019, representing 1 to 3rd of all global deaths.^[1]^ Atherosclerosis is undoubtedly the most frequent underlying cause of most CVDs. Hypercholesterolemia is a strong independent risk factor of atherosclerosis due to its ability to drive its establishment even when other known risk factors are absent.^[2]^

Interest in understanding cholesterol synthesis led to the discovery of competitive HMG-CoA reductase inhibitors, thus forming the first and most important class of mevalonate pathway inhibitors, statins.^[3]^ There are currently 7 commercial statins: atorvastatin, fluvastatin, lovastatin, pitavastatin, pravastatin, rosuvastatin, and simvastatin.^[4]^ In 2018, the American Heart Association/American College of Cardiology guidelines recommended statin therapy for all adults with low-density lipoprotein-C >190 mg/dL, all subjects with an established atherosclerotic cardiovascular disease, practically all patients with type 2 diabetes, and adults over 40 with predicted 10-year atherosclerotic cardiovascular disease risk ≥5% in the presence of other risk enhancing factors.^[4]^

On the other hand, cancer is the second leading cause of death after CVD.^[5]^ Cancer incidence and mortality are increasing worldwide. Two thousand twenty Globocan statistics showed an estimated number of new cancer cases of 19.3 million and an estimated number of cancer deaths of 8.2 million around the world.^[6]^ In Lebanon, 11,589 new cancer cases and 6,438 deaths were recorded in 2020, almost 3 times the cancer incidence during the past 2 decades. The top 5 most frequent cancers in Lebanon in 2020 were breast, lung, prostate, colorectal, and bladder cancer.^[7]^ One of the main features of cancer cells is altered metabolism. The mevalonate pathway is an essential metabolic pathway for all cells. Recent studies have found that cancer cells exhibit an increased demand for numerous mevalonate pathway intermediates that assist in tumor cell growth.^[8]^ Accordingly, there is increasing evidence concerning the anticancer effect of statins.^[9]^ Studies have shown a beneficial effect of several statins concerning the decrease of inflammatory markers,^[10]^ inhibition of angiogenesis,^[11]^ induction of apoptosis,^[12]^ and a decrease in tumor growth,^[13]^ and metastasis.^[14]^ Epidemiological studies were controversial since many of them showed a protective association between the intake of statins and cancer development,^[14–18]^ while others showed this association to be neutral,^[19–21]^ or harmful.^[22]^

Given these controversial data regarding the presence or absence of cancer risk in association with statin usage, we designed a study to determine the possible risk of cancer development associated with statin intake. In addition, this type of research related to this topic is inexistent in Lebanon. There is therefore a strong need for such precise scientific data. The main objective of this study was to determine if statin intake is associated with any protective, neutral, or harmful effect on lung, colorectal, or bladder cancers development in the Lebanese population and more specifically, to determine if the association is dependent on statin type, dose, and duration of use and if there is a correlation with gender, age, risk factors, or comorbidities. These objectives were also assessed within the setting of other risk factors and other medications commonly taken by our population.

## 2. Methods

### 2.1. Ethical aspects

Several steps were implemented in our study to ensure the participants confidentiality such as using numbers to label data instead of using names and keeping a separate list of number-to-name match-ups. This study has no potential risks since it is based on data collection from medical files, and no clinical trial was conducted. Data collection was started after obtaining approval from the thesis committee of the Faculty of Medical Sciences at the Lebanese University and the IRB committees of each of the Lebanese Hospital Geitaoui (code: 2021-IRB-011) and Mount Lebanon Hospital (code: Onc-2021-003). Missing data from the medical files were filled in by calling the patients and collecting information based on informed consent. This study was conducted in accordance with Good Clinical Practice ICH Section 3, and the principles laid down by the 18^th^ World Medical Assembly (Helsinki, 1964), and all applicable amendments.

### 2.2. Study design and population

We conducted a multicentric, retrospective, case-control study on Lebanese subjects from 2 Lebanese hospitals affiliated with the Lebanese University, Faculty of Medical Sciences: Lebanese Hospital Geitaoui and Mount Lebanon Hospital.

The minimum sample size was calculated using G-Power, with an effect size of 0.5, an alpha error of 5%, and a power of 95%. A total number of 339 was obtained, divided into 2 main groups: control (subjects with no cancer +/− statin intake; n = 135) and cases (subjects with either lung (n = 68), colorectal (n = 68), or bladder cancer (n = 68) +/− statin intake; n = 204). A total of 4 subgroups were obtained: group 1 (control group) consisting of cancer-free patients with no statin use, group 2 consisting of lung, colorectal, or bladder cancer patients with no statin use, group 3 consisting of cancer-free patients on statin therapy, and group 4 consisting of lung, colorectal, or bladder cancer patients taking statins.

Lebanese citizens between 30 and 100 years of age were included in our study. We selected lung, colorectal, and bladder cancer patients diagnosed between 2011 and 2021. Concerning group 4 (cancer subjects on statin therapy), we included those who have a minimum of 12 months of statin therapy preceding cancer diagnosis. However, subjects with primary cancer diagnosis before 12 months of statin therapy, those with primary cancer other than lung, colorectal, or bladder cancer, and those with insufficient history in the database were excluded from our study. No other restrictions were imposed in the selection of the population in this study.

### 2.3. Data collection

#### 2.3.1. Questionnaires and surveys

Data about the patients were collected using the hospitals medical files and filled on a Google form sheet from October 2019 till August 2021.

Our questionnaire included questions about the following: demographic information (gender, year of birth, work status, educational level, socioeconomic status, etc), biometrics and behavioral characteristics (height, weight, BMI, and smoking status), comorbidities (coronary artery disease, dyslipidemia, hypertension, diabetes mellitus, chronic kidney disease, etc), cancer status (no cancer, lung cancer, colorectal cancer [CRC], and bladder cancer), cancer type (for each of the lung, colorectal, and bladder cancers), cancer risk factors (age, gender, family history, smoking status, physical inactivity, occupational exposure, etc), statin type (atorvastatin, rosuvastatin, simvastatin, pravastatin, and pitavastatin), statin dosage, statin duration of use, and use of other medications [Aspirin, proton pump inhibitors (PPI), diuretics, metformin, angiotensin-converting enzyme inhibitors (ACEI), and fenofibrates].

#### 2.3.2. Work plan

The information needed for data collection such as the participants cancer status and the dose and duration of statin intake were retrieved from the patients medical files. When essential missing data were encountered, the patients were contacted via phone calls to fill in the required information. We entered data into Excel sheets for analysis and validated that all information collected was filled in correctly. Data analysis denoted categorizing variables and cross-tabulation, and we applied descriptive statistics and several statistical analysis tests and associations on them.

### 2.4. Data analysis

Data were analyzed using IBM SPSS version 25. Study variables were presented as per their type. Categorical variables were presented as frequency and proportions (example: gender, comorbidities, and risk factors). Continuous variables were presented as the mean, median, and standard deviation (example: duration of receiving the study treatments). Bivariate analysis was conducted to test the association between statins use (all statins and each molecule alone) and overall cancer development. The tests used were chi-square, Fisher exact test, and Mann–Whitney *U* test. Gender and age differences were assessed for lung cancer, CRC, and bladder cancer. In addition, we tested the association between statin intake and the development of each cancer type separately while controlling comorbid factors and risk/protective factors for each cancer. Testing was done between statin use and cancer development among populations where statins are frequently administered: hypertensive patients, diabetics, and patients with CVD. Bivariate and Multivariate analyses were also conducted to test the association between statin use and the development of each cancer type. In addition, we aimed to assess the association between the use of other medications (PPI, metformin, Aspirin, ACEIs, and fenofibrate) on cancer development, as the most encountered medications. Binary logistic analysis was enrolled to predict factors affecting each of the 3 cancer types where odds ratio (OR) with 95% confidence intervals was presented. Statistical significance was indicated at the 0.05 level.

## 3. Results

### 3.1. Demographics

Our sample contained a total of 709 patients meeting the inclusion criteria for this study. Among the 709 participants, 63.2% were males and 36.8% were females. A total of 51.9% were current smokers, 28.5% were diabetic, 59.9% were hypertensive, 26.9% suffered from CVD, and 31.2% were dyslipidemic. A proportion of 75% of our sample (531 cases) was cancer-positive, among which 24.1% had lung cancer, 26.7% had CRC, and 24.1% had bladder cancer (Table 1). Moreover, since cardiovascular patients are known to have several other comorbidities and will be on many other medications, hence we included the most encountered medications: Aspirin, PPIs, and metformin. It is worth noting that the high rate of missing values for BMI is attributed to the fact that a large number of patients could not remember their weight before getting diagnosed with cancer.

**Table 1:** General characteristics of the study population.

			Frequency	Percent
Gender	Male		448	63.2
	Female		261	36.8
Age	N		709	
	Mean (SD)		66.5 (11.5)	
	Median [min–max]		67 [27–98]	
BMI	N		596	
	Mean (SD)		26.9 (4.9)	
	Median [Min–Max]		26.2 [15.42–51.42]	
Smoking status	Non smoker		257	36.2
	Ex-smoker		84	11.8
	Current smoker		368	51.9
	Total		709	100.0
Cardiovascular disease	No		518	73.1
	Yes		191	26.9
	Average (yr): 8.8			
	Total		709	100.0
Diabetes	No		507	71.5
	Yes		202	28.5
	Average (yr): 11.7			
	Total		709	100.0
Dyslipidemia	No		488	68.8
	Yes		221	31.2
	Average (yr): 10.2			
	Total		709	100.0
Hypertension	No		284	40.1
	Yes		425	59.9
	Average (yr): 11.4			
	Total		709	100.0
Group	Control		178	25.1
	Cancer	Total	531	74.9
		Lung	171	24.1
		Bladder	171	24.1
		Colorectal	189	26.7
	Total		709	100

Data are mean (SD) [Minimum-Maximum] for quantitative variables or percent for categorical. Average (years) represents the number of years the subject has been suffering from each preexisting medical condition, out of those who answered “Yes”.

BMI = body mass index (Kg/m^2^), SD = standard deviation.

### 3.2. Statins and cancer development

Among the included subjects, 32% of cancer patients (170 among 531 individuals) and 32% of the control group (57 among 178 individuals) were statins users. Our study showed no significant associations between statins use and overall cancer development (Table 2). After analyzing statins use in each cancer type alone, all statins use was significantly associated with lung cancer (harmful) (OR = 1.823; *P* value = .007), atorvastatin with lung cancer (harmful) (OR = 1.729; *P* value = .034), and atorvastatin with bladder cancer (protective) (OR = 0.471; *P* value = .019) (Table 2). No significant association was found between dose and duration of each statin molecule and each cancer type (Table 3).

**Table 2:** Bivariate analysis between Statins use and cancer development.

			Group		Total	*P* value	OR (95% confidence interval)
			Control	Cancer			
Overall cancer development	Statins use	No	121	361	482	.999	1.000 (0.695–1.438)
			68.0%	68.0%	68.0%		
		Yes	57	170	227		
			32.0%	32.0%	32.0%		
	Atorvastatin	No	146	440	586	.798	0.944 (0.605–1.471)
			82.0%	82.9%	82.7%		
		Yes	32	91	123		
			18.0%	17.1%	17.3%		
	Rosuvastatin	No	159	482	641	.571	0.851 (0.486–1.488)
			89.3%	90.8%	90.4%		
		Yes	19	49	68		
			10.7%	9.2%	9.6%		
	Simvastatin	No	174	507	681	.178	2.059 (0.705–6.018)
			97.8%	95.5%	96.1%		
		Yes	4	24	28		
			2.2%	4.5%	3.9%		
Lung cancer	Statins use	No	121	92	213	.007	1.823 (1.179–2.817)
			68.0%	53.8%	61.0%		
		Yes	57	79	136		
			32.0%	46.2%	39.0%		
	Atorvastatin	No	146	124	270	.034	1.729 (1.040–2.877)
			82.0%	72.5%	77.4%		
		Yes	32	47	79		
			18.0%	27.5%	22.6%		
	Rosuvastatin	No	159	148	307	.426	1.300 (0.681–2.485)
			89.3%	86.5%	88.0%		
		Yes	19	23	42		
			10.7%	13.5%	12.0%		
	Simvastatin	No	174	162	336	.164	2.417 (0.730–8.000)
			97.8%	94.7%	96.3%		
		Yes	4	9	13		
			2.2%	5.3	3.7%		
Colorectal cancer	Statins use	No	121	139	260	.241	0.764 (0.486–1.199)
			68.0%	73.5%	70.8%		
		Yes	57	50	107		
			32.0%	26.5%	29.2%		
	Atorvastatin	No	146	161	307	.413	0.793 (0.456–1.381)
			82.0%	85.2%	83.7%		
		Yes	32	28	60		
			18.0%	14.8%	16.3%		
	Rosuvastatin	No	159	176	335	.198	0.618 (0.296–1.292)
			89.3%	93.1%	91.3%		
		Yes	19	13	32		
			10.7%	6.9%	8.7%		
	Simvastatin	No	174	184	358	.805	1.182 (0.312–4.474)
			97.8%	97.4%	97.5%		
		Yes	4	5	9		
			2.2%	2.6%	2.5%		
Bladder cancer	Statins use	No	121	130	251	.095	0.670 (0.418–1.073)
			68.0%	76.0%	71.9%		
		Yes	57	41	98		
			32.0%	24.0%	28.1%		
	Atorvastatin	No	146	155	301	.019	0.471 (0.248–0.894)
			82.0%	90.6%	86.2%		
		Yes	32	16	48		
			18.0%	9.4%	13.8%		
	Rosuvastatin	No	159	158	317	.320	0.689 (0.329–1.442)
			89.3%	92.4%	90.8%		
		Yes	19	13	32		
			10.7%	7.6%	9.2%		
	Simvastatin	No	174	161	335	.105	2.702 (0.831–8.786)
			97.8%	94.2%	96.0%		
		Yes	4	10	14		
			2.2%	5.8%	4.0%		

OR = odds ratio.

**Table 3:** Bivariate analysis between duration and dose of statin use and each cancer type.

			N	Mean	Std. Deviation	Minimum	Maximum	*P* value
Lung								
Dose of atorvastatin (mg)	Control		76	22.17	10.87	5	40	.619
	Lung cancer		47	22.55	10.73	10	40	
	Total		123	22.32	10.78	5	40	
Duration of use of atorvastatin (yr)	Control		76	9.41	5.49	0	20	.420
	Lung cancer		47	8.02	5.47	0	20	
	Total		123	8.88	5.50	0	20	
Dose of rosuvastatin (mg)	Control		45	17.44	13.21	5	80	.831
	Lung cancer		23	15.00	9.17	5	40	
	Total		68	16.62	11.98	5	80	
Duration of use of rosuvastatin (yr)	Control		45	9.53	5.04	1	22	.740
	Lung cancer		23	8.61	5.85	1	20	
	Total		68	9.22	5.30	1	22	
Dose of simvastatin (mg)	Control		19	22.11	10.32	10	40	.754
	Lung cancer		9	22.22	6.67	20	40	
	Total		28	22.14	9.17	10	40	
Duration of use of simvastatin (yr)	Control		19	9.74	6.21	1	21	.386
	Lung cancer		9	8.89	4.14	2	15	
	Total		28	9.46	5.56	1	21	
Colorectal								
Dose of atorvastatin (mg)		Control	95	22.74	10.96	10	40	.320
		Colorectal cancer	28	20.89	10.19	5	40	
		Total	123	22.32	10.78	5	40	
Duration of use of atorvastatin (yr)		Control	95	8.89	5.62	0	20	.940
		Colorectal cancer	28	8.86	5.15	1	20	
		Total	123	8.88	5.50	0	20	
Dose of rosuvastatin (mg)		Control	55	15.55	9.21	5	40	.300
		Colorectal cancer	13	21.15	19.81	5	80	
		Total	68	16.62	11.98	5	80	
Duration of use of rosuvastatin (yr)		Control	55	9.04	5.46	1	22	.203
		Colorectal cancer	13	10.00	4.67	5	21	
		Total	68	9.22	5.30	1	22	
Dose of simvastatin (mg)		Control	23	23.04	9.74	10	40	.661
		Colorectal cancer	5	18.00	4.47	10	20	
		Total	28	22.14	9.17	10	40	
Duration of use of simvastatin (yr)		Control	23	10.13	5.71	2	21	.902
		Colorectal cancer	5	6.40	3.91	1	10	
		Total	28	9.46	5.56	1	21	
Bladder								
Dose of atorvastatin (mg)		Control	107	22.48	10.63	5	40	.332
		Bladder cancer	16	21.25	12.04	10	40	
		Total	123	22.32	10.78	5	40	
Duration of use of atorvastatin (yr)		Control	107	8.50	5.31	0	20	.227
		Bladder cancer	16	11.44	6.21	3	20	
		Total	123	8.88	5.50	0	20	
Dose of rosuvastatin (mg)		Control	55	16.18	12.32	5	80	.200
		Bladder cancer	13	18.46	10.68	10	40	
		Total	68	16.62	11.98	5	80	
Duration of use of rosuvastatin (yr)		Control	55	8.60	4.92	1	21	.038
		Bladder cancer	13	11.85	6.23	2	22	
		Total	68	9.22	5.30	1	22	
Dose of simvastatin (mg)		Control	18	21.11	7.58	10	40	.818
		Bladder cancer	10	24.00	11.74	10	40	
		Total	28	22.14	9.17	10	40	
Duration of use of simvastatin (yr)		Control	18	7.67	4.04	1	15	.135
		Bladder cancer	10	12.70	6.62	4	21	
		Total	28	9.46	5.56	1	21	

### 3.3. The effect of cancer-specific risk factors and comorbidities on lung cancer, CRC, and bladder cancer development using bivariate analysis

Our results showed a statistically significant male predominance for lung cancer among cancer patients with a *P* value of <.001 (69.6% males vs 30.4% females). Among the known lung cancer risk factors (smoking, pollution including asbestos exposure, family history of lung cancer, and chest irradiation), only smoking was found to significantly increase the risk of lung cancer development (OR = 9.114; *P* value <.001). As for the rest of lung cancer risk factors, no significant correlation with lung cancer development was established (Table 4).

**Table 4:** Bivariate analysis between different risk factors and each cancer type.

Lung cancer			Control		Lung cancer		Total	*P* value
Gender	Male		94		119		213	.001
			52.8%		69.6%		61.0%	
	Female		84		52		136	
			47.2%		30.4%		39.0%	
Risk factors	Control		Cancer	Total	*P* value		OR (95% confidence interval)	
Smoking	No	86	16	102	.000		9.114 (5.039–16.484)	
		48.3%	9.3%	29.1%				
	Yes	92	156	248				
		51.7%	90.7%	70.9%				
Pollution including asbestos exposure	No	20	17	37	.695		1.147 (0.579–2.272)	
		11.2%	9.9%	10.6%				
	Yes	158	154	312				
		88.8%	90.1%	89.4%				
Family history of lung cancer	No	178	170	348	.149		0.489 (0.439–0.544)	
		100.0%	98.8%	99.4%				
	Yes	0 0.0%	2 1.2%	2 0.6%				
CRC		Control	Cancer	Total	*P* value		OR (95% confidence interval)	
Age more than 50	No	22	19	41	.472		1.269 (0.662–2.434)	
		12.4%	10.0%	11.1%				
	Yes	156	171	327				
		87.6%	90.0%	88.9%				
Personal history of IBD, colorectal cancer and or polyps	No	175	179	354	.088		3.259 (0.882–12.041)	
		98.3%	94.7%	96.5%				
	Yes	3	10	13				
		1.7%	5.3%	3.5%				
Family history of colorectal cancer	No	177	174	351	.001		15.259 (1.994–116.766)	
		99.4%	92.1%	95.6%				
	Yes	1	15	16				
		0.6%	7.9%	4.4%				
Smoking	No	86	102	188	.279		0.797 (0.529–1.202)	
		48.3%	54.0%	51.2%				
	Yes	92	87	179				
		51.7%	46.0%	48.8%				
Alcohol	No	157	172	329	.378		0.739 (0.376–1.451)	
		88.2%	91.0%	89.6%				
	Yes	21	17	38				
		11.8%	9.0%	10.4%				
Diet: red meat, unhealthy food	No	49	48	97	.644		1.116 (0.701–1.775)	
		27.5%	25.4%	26.4%				
	Yes	129	141	270				
		72.5%	74.6%	73.6%				
Overweight	No	49	49	98	.132		0.689 (0.424–1.120)	
		29.2%	37.4%	32.8%				
	Yes	119	82	201				
		70.8%	62.6%	67.2%				
Physical inactivity	No	51	72	123	.055		0.653 (0.421–1.011)	
		28.7%	38.1%	33.5%				
	Yes	127	117	244				
		71.3%	61.9%	66.5%				
Bladder cancer			Control	Cancer	Total	*P* value	OR (95% confidence interval)	
Age more than 50		No	22	13	35	.134	1.725 (0.839–3.545)	
			12.4%	7.6%	10.0%			
		Yes	156	159	315			
			87.6%	92.4%	90.0%			
Male		No	84	41	125	.000	2.855 (1.806–4.513)	
			47.2%	23.8%	35.7%			
		Yes	94	131	225			
			52.8%	76.2%	64.3%			
Personal history of chronic bladder infection and or stones or polyps		No	166	154	320	.213	1.617 (0.754–3.466)	
			93.3%	89.5%	91.4%			
		Yes	12	18	30			
			6.7%	10.5%	8.6%			
Family history of bladder cancer		No	177	168	345	.208	4.214 (0.466–38.089)	
			99.4%	97.7%	98.6%			
		Yes	1	4	5			
			0.6%	2.3%	1.4%			
Smoking		No	86	55	141	.002	1.989 (1.287–3.072)	
			48.3%	32.0%	40.3%			
		Yes	92	117	209			
			51.7%	68.0%	59.7%			
Occupational exposure (aniline dyes, leather, rubber and paint)		No	175	169	344	1.000	1.036 (0.206–5.202)	
			98.3%	98.3%	98.3%			
		Yes	3	3	6			
			1.7%	1.7%	1.7%			
Pelvic irradiation		No	176	168	344	.442	2.095 (0.379–11.590)	
			98.9%	97.7%	98.3%			
		Yes	2	4	6			
			1.1%	2.3%	1.7%			

CRC = colorectal cancer, OR = odds ratio.

Among CRC risk factors (age more than 50, personal history of inflammatory bowel disease or polyps, family history of CRC, smoking, alcohol intake, a diet rich in red meat, being overweight, and physical inactivity), only family history was found to be significantly associated with the development of CRC (OR = 15.259; *P* value <.001) (Table 4).

Among bladder cancer patients, 92.4% were above 50 years of age, in contrast with 87.6% of the control group. However, age above 50 was not proven to be significantly associated with the development of bladder cancer in our study (*P* value =.13). A proportion of 76.2% of bladder cancer patients vs 52.8% of the control group were males, which established a significant association of the male gender with the development of bladder cancer (OR = 2.86; *P* value <.001). Among the remaining bladder cancer risk factors, smoking was shown to be significantly associated with developing bladder cancer (OR= 1.989; *P* value <.01) (Table 4).

### 3.4. The effect of other medications use on lung cancer, CRC, and bladder cancer development using bivariate analysis

The use of Aspirin was found to be positively correlated (harmful association) with lung cancer incidence with a *P* value = .04 (OR = 1.59), and negatively correlated (protective association) with CRC incidence with a *P* value <.001 (OR = 0.45). On the other hand, PPIs intake was significantly associated with an increased lung cancer incidence (OR = 2.87), and decreased CRC incidence (OR = 0.31). Meanwhile, the use of metformin was shown to have a negative correlation (protective association) with the development of each of lung (OR = 0.51), colorectal (OR = 0.36), and bladder cancer (OR = 0.46). The use of ACEIs was found to be positively correlated with lung (*P* value = .014; OR = 4.403) and bladder cancer (*P* value = .002; OR = 6.022) but negatively correlated with CRC (*P* value = .001; OR = 0.363). Fenofibrate use was not significantly associated with any cancer type development (Table 5).

**Table 5:** Other medications used by our study population.

		Control	Lung cancer	CRC	Bladder cancer
Aspirin	No	125	102	159	135
		70.2%	59.6%	84.1%	78.9%
	Yes	53	69	30	36
		29.8%	40.4%	15.9%	21.1%
	*P* value		.038	.001	.062
	OR (95% confidence interval)		1.595 (1.024–2.486)	0.445 (0.268–0.738)	0.629 (0.386–1.025)
PPI	No	144	102	176	151
		80.9%	59.6%	93.1%	88.3%
	Yes	34	69	13	20
		19.1%	40.4%	6.9%	11.7%
	*P* value		.000	.000	.056
	OR (95% confidence interval)		2.865 (1.768–4.643)	0.313 (0.159–0.615)	0.561 (0.309–1.020)
Metformin	No	138	149	171	151
		77.5%	87.1%	90.5%	88.3%
	Yes	40	22	18	20
		22.5%	12.9%	9.5%	11.7%
	*P* value		.019	.001	.008
	OR (95% confidence interval)		0.509 (0.288–0.900)	0.363 (0.199–0.662)	0.457 (0.255–0.820)
ACEIs	No	175	159	171	155
		98.3%	93.0%	90.5%	90.6%
	Yes	3	12	18	16
		1.7%	7.0%	9.5%	9.4%
	*P* value		.014	.001	.002
	OR (95% confidence interval)		4.403 (1.220–15.886)	0.363 (0.199–0.662)	6.022 (1.722–21.058)
Fenofibrate	No	172	164	186	167
		96.6%	95.9%	88.4%	97.7%
	Yes	6	7	3	4
		3.4%	4.1%	1.6%	2.3%
	*P* value		.783	.325	.751
	OR (95% confidence interval)		1.224 (0.403–3.717)	0.462 (0.114–1.877)	0.687 (0.190–2.477)

CRC = colorectal cancer, OR = odds ratio.

### 3.5. Multivariate analysis

After assessing for all potential confounding factors affecting the development of each of the cancer types using multivariate analysis, a significantly positive correlation (harmful association) remained between lung cancer development on 1 side and each of PPIs use (OR= 3.42; (1.929–6.067), *P* value< .001), smoking (OR = 10.57), and duration of simvastatin intake (OR = 1.208 (1.002–1.457), *P* value = .048). No significant association was found between each of atorvastatin and rosuvastatin and lung cancer. Multivariate analysis also revealed a positive association (harmful association) between CRC development and: age (above 50, OR = 3.88), family history of CRC (OR = 14.52), and physical inactivity (OR = 2.02); and a negative correlation (protective association) between the development of this type of cancer and PPIs intake (OR = 0.38 (0.167–0.869), *P* value = .022). No significant association was found between each statin and CRC. Regarding bladder cancer, we found a significant positive correlation (harmful association) between its incidence and age (OR = 3.01), male gender (OR = 3.57), smoking (OR = 1.73), and increased duration of simvastatin intake (OR = 1.189 (1.028–1.376), *P* value = 0.020) (Table 6). No significant association was found between each of atorvastatin and rosuvastatin and lung cancer.

**Table 6:** Multivariate analysis concerning the effect of several factors on cancer development.

	B	S.E.	df	*P* value	OR (95% CI)
Lung cancer					
Duration of use of simvastatin (yr)	0.189	0.096	1	.048	1.208 (1.002–1.457)
PPI	1.230	0.292	1	.000	3.421 (1.929–6.067)
Smoking	2.358	0.342	1	.000	10.572 (5.406–20.673)
CRC					
Age (cutoff 50)	1.355	0.476	1	.004	3.875 (1.525–9.849)
Family history of CRC	2.676	1.114	1	.016	14.521 (1.635–128.995)
Physical inactivity	0.702	0.314	1	.025	2.018 (1.091–3.731)
PPI	-0.967	0.421	1	.022	0.380 (0.167–0.869)
Bladder cancer					
Male gender	1.273	0.264	1	.000	3.572 (2.128–5.995)
Smoking	0.548	0.254	1	.031	1.729 (1.051–2.845)
Duration of use of simvastatin (yr)	0.173	0.074	1	.020	1.189 (1.028–1.376)
Age (cutoff 55)	1.103	0.345	1	.001	3.014 (1.534–5.921)

Data are mean (SD) [minimum-maximum] for quantitative variables or percent for categorical.

B = coefficient ß, CI = confidence interval, CRC = colorectal cancer, df = degrees of freedom, PPI = proton pump inhibitors, OR = odds ratio, S.E. = standard error.

## 4. Discussion

**Figure 1:**
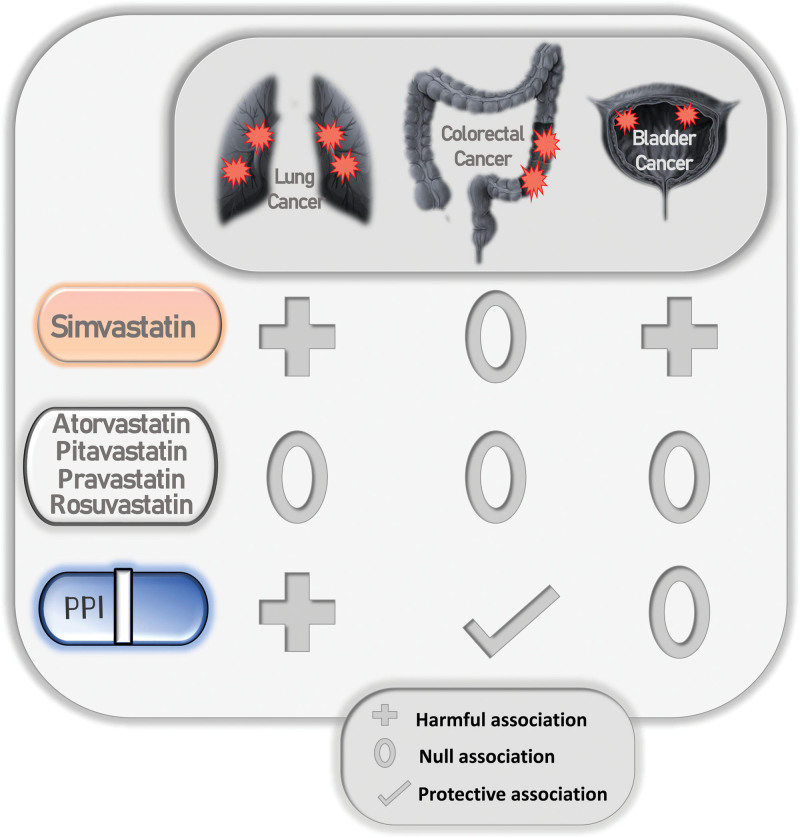
Protective vs Harmful association between different medications use such as Simvastatin, other statins, PPIs, and the incidence of lung, colorectal, and bladder cancer. PPI = proton pump inhibitors.

In our study, no significant association was established between the overall intake of statins and the risk of lung, colorectal, and bladder cancer development. This null association was confirmed after assessing for all confounding factors. However, after including the duration of statin intake in our multivariate analyses, we found that a longer duration of simvastatin was significantly associated (harmful) with the development of lung (OR= 1.208) and bladder cancer (OR = 1.189) (Table 6, Fig. 1).

Our results agree with a series of case-control studies including 88,125 cases and 362,254 matched controls reviewed by Vinogradova Y. et al^[22]^ who suggested that the use of statins does not affect the overall risk of cancer. However, when they examined each cancer type independently, they also found a significantly increased risk of lung cancer development (OR = 1.18), bladder cancer development (OR = 1.29), and colon cancer development (OR = 1.23) associated with prolonged statin intake (more than 4 years).

Nevertheless, the results of our study contradict the most recently presented evidence in the literature suggesting that statins, namely simvastatin, offer a potential protective effect against the development of several types of cancer, and may be candidates for future implementation in cancer prevention and treatment. Several experimental studies showed a protective effect of simvastatin against lung cancer incidence by inducing lung cancer cell apoptosis,^[15]^ decreasing its viability in a time and dose-dependent manner,^[16]^ and inhibiting tumor cell proliferation and bone metastasis.^[14]^ In addition, other studies suggested the possibility that simvastatin is protective against bladder cancer development by inducing cell cycle arrest, inhibition of bladder cancer cells proliferation, and suppression of bladder cancer cells metastasis.^[17]^ Moreover, Liu JC. et al^[18]^ found that the adjusted hazard ratio for colon cancer is decreased in statins users in general, with a stronger preventive effect in hydrophilic statin users such as simvastatin (adjusted hazard ratio = 0.52, 95% CI).

On the other hand, some studies in the literature showed no significant association between statin intake, specifically simvastatin and atorvastatin, and the development of lung, prostate, skin, and breast cancer.^[21]^ In addition, a meta-analysis conducted by Zhang XL. et al^[20]^ found no significant evidence associating statin intake with an increased risk of development of bladder cancer among daily statin users compared to nonusers. In their meta-analysis about statins and cancer risk, Dale et al^[19]^ found no type of cancer (colon, breast, prostate, respiratory, gastrointestinal, or melanoma) being affected by statin use and no subtype of statin affecting the risk of cancer development.

Furthermore, our results concerning lung, colorectal, and bladder cancer risk factors are in line with the literature. For instance, we found male gender and smoking to be correlated with lung cancer development, which is supported by Temraz, S. et al^[23]^ and GLOBOCAN statistics.^[7]^ Concerning CRC, our results suggest that positive family history increases the risk of developing this type of cancer are consistent with the literature. As for bladder cancer, our results are in line with the known bladder cancer risk factors such as male gender and smoking.^[6]^

### 4.1. Other medications potential effects on lung, colorectal, and bladder cancer

Other findings include the potential risk the intake of Aspirin, PPIs, and metformin has on the development of lung, colorectal, and bladder cancer. However, the duration of therapy was not taken into consideration. Our bivariate analysis showed a positive association correlation (harmful association) between Aspirin intake and an increased lung cancer incidence, in parallel with those of Dong X. et al^[24]^ In contrast, we found a potential protective effect of Aspirin intake against CRC development, in line with a meta-analysis conducted by Harewood R. et al^[25]^ As for PPI therapy, as shown in our multivariate analysis, we found a positive association correlating it with an increased lung cancer incidence (Table 6, Fig. 1), which was supported by Dresler H. et al^[26]^ results. On the other side, there was a significant decrease in CRC cases among PPI users in our study. Few experimental studies supported our findings,^[27]^ however, others found this correlation to be positive correlation (harmful association),^[28]^ which raises the need for further studies linking or not the intake of PPIs to the development of CRC. Furthermore, the incidence of lung, colorectal, and bladder cancer in metformin users among our participants was found to be decreased in our bivariate analysis. In fact, a meta-analysis suggested a protective effect of metformin intake against lung cancer^[29]^ and CRC development.^[30]^ Meanwhile, metformin intake failed to be associated with a decreased incidence of bladder cancer, however, it was found to improve the prognosis of bladder cancer patients in a large meta-analysis.^[31]^Therefore, clinical trials are necessary to uncover the possible effect of this molecule on the incidence of different types of cancer.

### 4.2. Limitations and strengths

Some limitations of our study should be recognized. First, the data for our retrospective study were collected from patients medical files at the hospitals, and missing data concerning the exact period of statin use and the duration of comorbidities were filled via phone calls, which raised the issue of recall bias. Second, our sample size was small (709 participants) relatively to that of studies in the literature, which makes subclassification of variables difficult. In addition, the number of patients taking simvastatin in our sample was limited (3.9% of the total number of participants), which was not sufficient to establish a causality relationship. Third, we were not able to establish a dose-response relationship. Causality was also difficult to prove since the sample size of each one of the statins was relatively small. Fourth, the correlation between statin intake and the development of cancer in relation to the gender, age, and comorbidities was not established, necessitating future investigations with a larger sample. Finally, pollution could not be easily assessed as a risk factor for lung cancer. It was worth discussing, but no defined scale was used for categorization.

On the other hand, the strengths of this study are worthy to note. To start with, this is the first study in Lebanon and the Middle East discussing the effect of statin intake on lung, colorectal, and bladder cancer development. Second, the time-response relationship was tested in our study. Data concerning the duration of statin intake and the corresponding risk of cancer development was collected and analyzed. Also, data such as co-medications (Aspirin, PPIs, metformin, ACEI, diuretics, and fenofibrate) were collected and checked for possible protective or harmful effect on cancer development. Further, our sample size (709 participants) is appropriate relatively to the small Lebanese population size. Therefore, our study may be considered as a future reference given that our results get validated to establish causality.

## 5. Conclusion

This study showed no significant association between statin therapy in general and the incidence of lung, colorectal, and bladder cancer. However, a time-dependent harmful association between simvastatin and lung and bladder cancer was detected. Statins are lifelong drugs; therefore, long-term side effects should be addressed before prescribing them to patients. Other drugs and specific risk factors were also associated negatively or positively with the development of these 3 cancer types. Being the only Lebanese study conducted on this topic, it may be considered a reference for physicians when choosing the type of statin to prescribe. However, the significance of our results and causality needs to be validated in future studies to establish a clearer link between the protective or harmful effects of statins and cancer risk, as this may influence many cancer patients’ decisions making.

## Acknowledgment

We are most grateful to Lebanese Hospital Geitaoui (LHG) and Mount Lebanon Hospital (MLH) who provided us with their list of patients. We thank the patients who kindly accepted to respond to our questions when data were missing from their files. We thank Prof. Roland Asmar for providing his input on the initial idea of this article.

## Author contributions

**Conceptualization:** Issam G. Chalhoub, Rita T. Boulos, Karen G. Haifa, Issam Kassab, Mirna N. Chahine.

**Data curation:** Issam G. Chalhoub, Rita T. Boulos, Yara G. Dagher, Sandra El Helou, Bachir Atallah, Issam Kassab, Mirna N. Chahine.

**Formal analysis:** Bachir Atallah.

**Investigation:** Issam G. Chalhoub, Rita T. Boulos, Yara G. Dagher, Sandra El Helou, Karen G. Haifa, Mirna N. Chahine.

**Methodology:** Bachir Atallah, Issam Kassab, Mirna N. Chahine.

**Project administration:** Issam Kassab, Mirna N. Chahine.

**Resources:** Fadi Nasr.

**Supervision:** Issam Kassab, Mirna N. Chahine.

**Validation:** Fadi Nasr, Issam Kassab, Mirna N. Chahine.

**Visualization:** Mirna N. Chahine.

**Writing – original draft:** Issam G. Chalhoub, Rita T. Boulos.

**Writing – review & editing:** Issam Kassab, Mirna N. Chahine.
